# An “In-Situ Binding” Approach to Produce Torrefied Biomass Briquettes

**DOI:** 10.3390/bioengineering6040087

**Published:** 2019-09-20

**Authors:** Osama Bu Aamiri, Rajeeva Thilakaratne, Jaya Shankar Tumuluru, Jagannadh Satyavolu

**Affiliations:** 1 Conn Center for Renewable Energy Research, University of Louisville, Louisville, KY 40292, USA; Osama.Buaamiri@louisville.edu (O.B.A.); Chamila.Thilakaratne@louisville.edu (R.T.); 2 750 MK Simpson Boulevard, Energy Systems Laboratory, Idaho National Laboratory, Idaho Falls, ID 83415, USA; Jayashankar.tumuluru@inl.gov

**Keywords:** biomass briquetting, highly and lightly torrefied materials, lignin, binding, moisture, hydrophobicity, durability

## Abstract

Biomass-derived coal or “biocoal” produced using a torrefaction process presents a carbon-neutral option of coal for power generation. While torrefaction delivers a carbon content and hydrophobicity comparable to coal, it lowers its density and creates material handling, storage, and transportation challenges. Densification into briquettes would help mitigate these challenges. However, the torrefied biomass is difficult to densify and may require the use of binders, which are expensive and can be incompatible with respect to material and emissions. A cost-effective approach to utilize lignin in-situ of the biomass to promote binding during densification was demonstrated using a pilot-scale briquetter unit during this study. Lignin, a cross-linked polymer, tends to break down and lose its binding ability under high-temperature conditions of torrefaction. In this paper, we investigated the use of a lightly torrefied material as a binder―LTM (biomass torrefied in the transition region of non-reactive and reactive temperature ranges of torrefaction). When mixed with torrefied biomass and densified together under suitable moisture and temperature conditions, the lignin is shown to mobilize and provide binding to the briquettes. The results showed that briquettes produced using LTM as binder and 10% to 11% moisture provided in-situ binding, improved density and durability, and produced hydrophobic briquettes.

## 1. Introduction

Lignocellulosic biomass plays a significant role as a sustainable starting material in the production of renewable energy. However, some key challenges associated with the use of biomass in fuel applications are its low-energy density, non-isotropy, and poor storage stability (due to high-moisture uptake and low hydrophobicity). As a result, wood has not become a drop-in replacement for coal for electricity and power generation. Pre-treatment technologies (such as biomass torrefaction) have been developed to upgrade biomass, overcome its limitations, and raise its potential to substitute for coal [1,2,3,4,5]. Torrefaction is a thermal treatment process carried out in a chemically inert environment to improve the hydrophobicity and energy content of biomass. Torrefaction can be performed using several biomass types, such as wood waste, forest residues, agricultural wastes, and municipal solid wastes. The torrefaction temperature and time or duration are the key factors in determining the energy density and hydrophobicity of the torrefied biomass [6,7,8]. The resulting product is energy densified and appears as a brown-to-black solid, and the characteristics obtained after torrefaction strongly depend on process conditions, such as temperature, time of torrefaction, and type of feedstock, as well as the type of torrefaction equipment employed. Torrefaction makes biomass brittle and reduces the grinding energy by 70% to 90% compared to raw biomass and improves the particles’ size and shape characteristics. The chemical composition in terms of proximate and ultimate composition also improves after torrefaction [9]. 

Despite these advantages, several challenges arise after torrefaction of biomass materials, such as poor density and a high amount of dust formation during handling, which must be addressed for the commercial use of torrefied material. Rodrigues and Rousset [10] mentioned that the bulk density of *Eucalyptus* wood was reduced by 14.12% after been torrefied at 280 °C for 30 min. To make use of torrefied biomass commercially, the material must be densified. Densification is the process of compaction of torrefied materials or biomass residues into a product of higher density than the original raw material. Such a conversion has many advantages that include increasing calorific value per unit volume, using the convenience of transportation and storage, solving the problem of residue disposal, and making a uniform final product [11,12]. 

The challenge associated with the densification of torrefied biomass is the difficulty of getting the desirable binding to achieve density and durability of the briquettes. The main reason is the thermal degradation that occurs in the biomass components, such as lignin during torrefaction. During torrefaction, the temperature range of 50 °C to 150 °C is called a non-reactive temperature regime where mostly moisture in the biomass is lost [9]. In the temperature range of 150 °C to 200 °C (called the reactive temperature regime), there will be mild changes in the biomass chemical composition. The temperature regime of 200 °C to 300 °C, which is called a destructive regime, results in a significant change of the chemical composition mainly due to devolatilization and carbonization reactions. It was mentioned in the work of Nanou et al. [13] that during the destructive regime of torrefaction (200–300 °C), lignin undergoes reactions of depolymerization, demethoxylation, bond cleavage, and condensation, while hemicellulose decomposes. 

Brodin [14] studied the thermal behavior of lignin, a cross-linked polymer in biomass. Both linear and cross-linked polymers soften and become rubbery when temperatures reach the glass transition temperature (T_g_). In the glass region, a linear and a cross-linked polymer behave differently. A cross-linked polymer, such as lignin, does not melt with further heating and starts to decompose. A linear polymer, on the other hand, starts to melt, obtains flow properties, and flows readily. During torrefaction in the temperature range between the non-reactive and reactive temperature regime, it is highly likely that lignin softens, and can be forced to mobilize/displace under pressure, temperature and steam, while retaining its binding ability. In the high end of reactive and destructive regions (i.e., such as high-torrefaction temperatures), lignin breaks down and loses its ability to bind, which leads to poor densification and lower durability for the highly torrefied biomass. 

Different densification methods of the torrefied biomass have been proposed and investigated in the literature with the purpose of providing the necessary binding to the torrefied biomass. Some worked on increasing processing pressure, moisture content, and/or temperature, while others focused on preconditioning or using a binder, such as crude glycerol, gelatinized starch, lignosulfonate, molasses, and heavy pyrolysis oil [15,16,17]. Pellets and briquettes are the two forms of densified biomass available. Pellets are typically smaller in size (<1 cm diameter), while the briquettes are larger (>3 cm diameter) and are denser than pellets. Previous work [18,19] stated that the density and hardness (durability) of torrefied pellets was consistently lower than the non-torrefied pellets, and that large compression force is needed for making pellets from highly torrefied biomass samples [20]. The briquetting of torrefied biomass is less expensive than pelleting due to low capital and operating and maintenance costs of briquetting equipment. In addition, briquette presses can handle larger particle sizes, wider moisture contents, and better feed handling [21]. The data on briquetting characteristics of torrefied biomass in a pilot scale mechanical briquettes press is scarce or not available. In this work, our primary focus is on pilot scale briquetting of the torrefied biomass.

Several types of different binders during densification have been explored in the literature to improve the quality of torrefied pellets [22,23,24,25,26]. While most widely used binders can increase the quality of torrefied biomass after densification, they are either expensive, or are a part of the food supply. In addition, binders, especially from synthetic sources, can lower the emissions rating for biocoal products. Peng et al. [15] also discussed using untreated sawdust as a natural binder and other binders, such as starch and lignin, to improve the quality of torrefied sawdust pellets. They concluded that the torrefied pellets prepared with binders have a lower bulk density than un-torrefied pellets but with a comparable hardness, and that the natural binders (in the un-torrefied biomass) reduced the heating value of the torrefied pellets. They did not discuss the hydrophobicity, which is expected to be low with the use of hydrophilic untreated biomass as a binder. Also, blending raw biomass with torrefied biomass can have an impact on the calorific value. 

According to Kaliyan [27], high-moisture content during densification can increase binding in biomass material by activating (softening) the natural binders within that material, such as lignin. Lignin, an amorphous material, would undergo plastic deformation at pressures and temperatures in the range of their glass transition temperatures. In addition, the high-moisture content in the biomass can lower the glass transition temperature of lignin, and with the application of pressure and temperature, the natural binder can be activated in the biomass while deforming the particles. Hence, maintaining a certain level of moisture content during densification is needed to promote binding within the briquette. Anna and Souza [28] showed scanning electron microscope (SEM) and transmission electron microscope (TEM) images in which lignin-rich particles can be seen migrating on the surface of a biomass cell walls with diameters of 5 µm to 10 µm after a thermo-chemical treatment. 

Binders added to torrefied biomass can cost about $0.5–1/lb, and if about 10–20 lb of binder are used per ton of biomass, then the binder cost contribution is $5 to $20 per ton plus equipment and labor costs, as well as its negative impact on the heating value. Accordingly, a cost-effective approach without using any added binders is needed to densify the torrefied biomass. The overall objective of the project is to use lignin “in-situ” of biomass as a binder during briquetting of the torrefied biomass. 

In the present research, we developed a novel approach of utilizing the lignin in-situ of the biomass through the use of lightly torrefied biomass (LTM) to promote binding in the highly torrefied biomass (HTM). The biomass delivering lignin for binding―a lightly torrefied material (LTM)―is torrefied at a lower temperature range (in the transition zone of non-reactive and reactive temperature ranges of torrefaction) while the other biomass component―a highly torrefied material (HTM)―is torrefied in the reactive and destructive temperature ranges of torrefaction. Another major improvement in the present study is the wood chips after torrefaction are used as such without further grinding. Since both LTM and HTM become friable after torrefaction, size reduction of the torrefied coarse and fine chips inherently happened during mixing; hence, no separate grinding was needed. This approach can have a significant impact on the biomass preprocessing cost as grinding of both woody and herbaceous biomass is a major cost in biomass preprocessing for biofuels production.

The specific objectives of this study were to (1) understand the role played by LTM as a binder to deliver in-situ lignin for binding during briquetting, and (2) understand the impact of moisture to promote the mobility of in-situ lignin, and to increase binding during the briquetting process and its impact on the briquette quality attributes, such as unit density, durability, and water uptake during storage. 

## 2. Materials and Methods

### 2.1. Materials 

The torrefied materials used in this study were sourced from Solvay Biomass Inc. Softwood fine chips were torrefied at temperature around 200 °C for 40 min and were used as lightly torrefied materials (LTMs), and the other same type but with coarse chips were torrefied at around 280 °C for 40 min and were utilized as highly torrefied materials (HTMs). The moisture content was measured using a Denver Instrument IR60Q-Germany moisture analyzer using a 1-g sample. Characterizations of feed materials are shown in Table 1. 

### 2.2. Method for Briquetting

**Figure 1:**
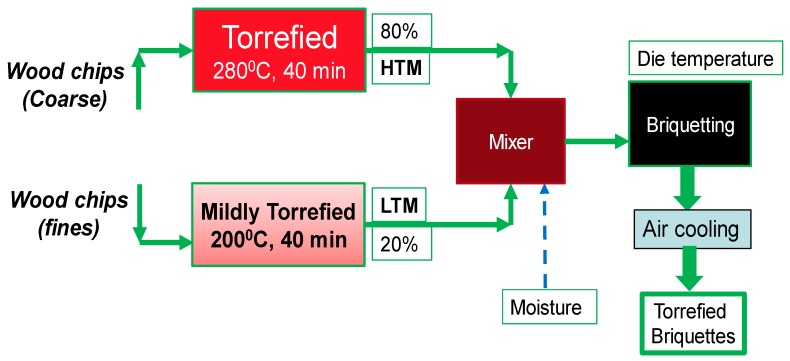
Process schematic to produce briquettes.

LTM as well as mixtures of LTM-HTM were prepared according to the experimental plan (Table 2). The briquetter machine specifications were (1) peak power at 12 KW, (2) piston speed at 5 in. per second, and (3) piston diameter of 36 mm. The process parameter tested during briquetting is the feed moisture content whereas other briquetting parameters, such as pressure, preheating temperature, die temperature, and feeding rate, were kept constant at 10,000 psi, 50 °C, 250 °C, and 10% respectively. A schematic for the briquettes production is shown in Figure 1.

**Figure 2:**
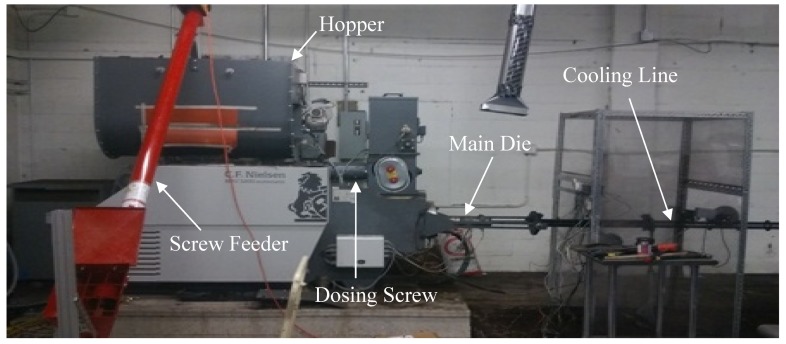
Pilot scale briquette press used in the present study.

Briquettes were produced using a C. F. Nielsen BPU 3200 pilot scale briquetter as illustrated in Figure 2. LTM material was loaded using a screw feeder. The HTM and LTM materials were mixed to a 20:80 weight ratio and their moisture content was adjusted as shown in Table 2. This mix ratio was selected based on our preliminary evaluations. A 100% LTM briquette was durable but not hydrophobic. A 100% HTM material did not form any briquettes. Hence, we started adding HTM material to LTM at 5% and 10%, which again showed durability but a lack of hydrophobicity. A 20% HTM addition provided both durability and hydrophobicity. 

**Figure 3:**
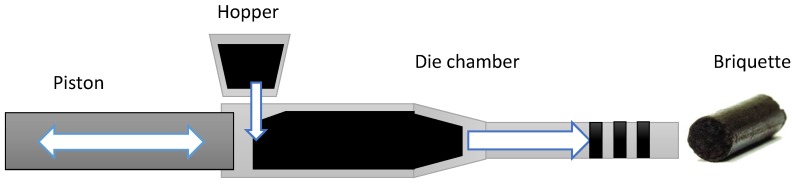
Schematic of briquette production process in a pilot scale briquette press.

**Figure 4:**
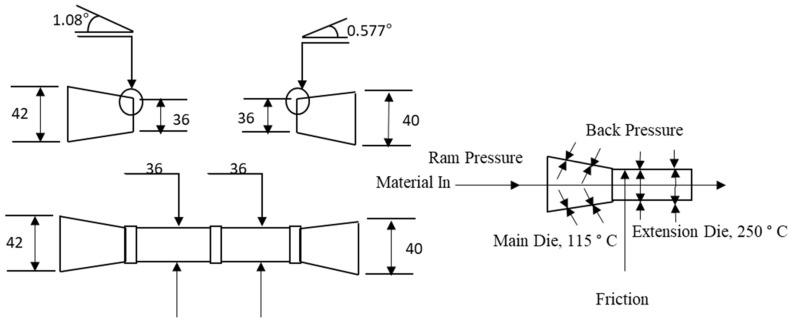
Die configuration schematic of pilot scale briquette press (diameter in mm).

**Figure 5:**
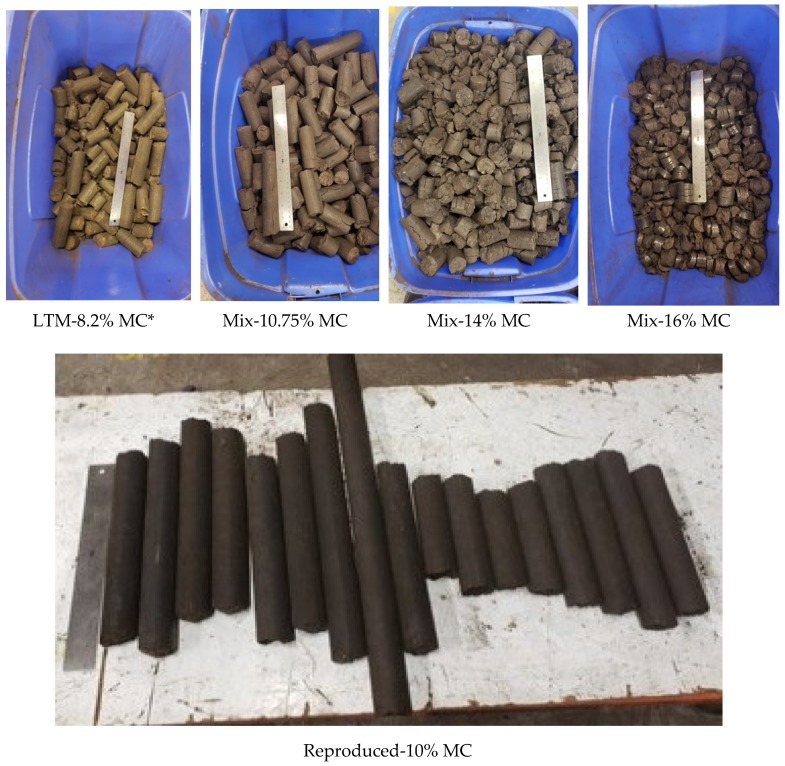
Produced briquettes. * MC: Moisture content (% w.b.).

The mixtures were preheated to about 50 °C by means of steam running through a copper coil. The die system in the briquetter was made from alloy steel and had three extensions after the main die. Different conical orifice angles for the main die and end extension were used. The results reported here were obtained using a die configuration with a 36-mm inner diameter. The die configuration was preheated to 250 °C and the material was fed from the main hopper through a dosing screw to the compression screw chamber at 10% of the rated feed capacity. The operating temperature of the die was 225 °C. During production, the temperature at the compression chamber was about 70 °C. The produced briquettes were further cooled to room temperature. The production rate of the briquettes produced at different moisture content was about 270–280 lb/hr, and the final moisture content in all briquettes varied from 6% to 8% (w.b.). Figure 2 and Figure 3 show the pilot scale briquette press and schematic of the briquetting process used in the present study. Figure 4 illustrates the die configuration schematic and angles used in the pilot scale briquette press. Figure 5 shows the briquettes produced at different moisture contents. 

### 2.3. Torrefied Briquette Physical Properties Characterization 

Unit density was calculated by using the water displacement method in which a small round sample of each briquette was taken after about 2 weeks of storage, and then weighed and coated with paraffin wax to prevent any water absorption during immersion in water. The waxed samples were weighed and then submerged into water. The volume of the displaced water was measured and recorded as the volume of the waxed sample. The volume of each sample was calculated by subtracting the volume of coating wax from the volume of waxed samples. The unit density of briquettes was calculated by dividing the weight of each sample by the volume [29]. 

Hydrophobicity, by means of moisture uptake, was measured using a Fischer Scientific Humidity Chamber device. The briquette samples were first weighed then placed in the humidity chamber and maintained in 90% humidity at 30 °C for 24 h. The samples were then weighed and dried in an oven at 60 °C for 6 h, and final weights were recorded. The change in weight refers to moisture absorption. A water immersion test was also conducted using two different times, 30 s and 30 min [30,31], to measure the water gain of each sample as an indication of hydrophobicity. Also, a water immersion test for 24 h was conducted to observe the qualitative effect of long-time direct water contact on the produced briquette’s shape. 

The durability index of produced briquettes was determined following an adopted method from ASAE S269.4 and ISO 15210 standards mainly used for pellet testing [32,33]. The mechanical durability of briquettes was tested by a specific abrasion drum (Garmet Automatic Sampling Equipment Company), in which samples of 500 ± 50 g were weighed to the nearest 0.1 g and placed in the tumbling box device. Then, they were tumbled at 50 ± 2 rpm for 500 rotations. Then, the samples were removed and sieved manually through a 3.15-mm mesh. The mechanical durability index (DU) was calculated using the following formula:

DU = (M_A_/M_E_) × 100, (1)

where M_E_ is the mass of the samples before the drum treatment and M_A_ is the mass of the sample left after sieving the resultant pieces after tumbling.

The calorific value of the prepared briquettes was measured using a bomb calorimeter type IKA C2000. The device was calibrated using benzoic tablets of 1 g and a sample of around 1 g was used from each briquette for calorific value analysis. 

The chemical functional groups that were present in raw and treated materials as well as produced briquettes were analyzed by Fourier transform infrared spectroscopy (FTIR) using powdered samples of about 20 mg from each. FTIR curves obtained were normalized to the C=C peak at 1506 cm^−1^ assuming negligible change in the aromatic ring count (originated from lignin) in the sample.

### 2.4. Data Analysis 

Many researchers have used regression models to understand the impact of the process variables on biomass commination and pelleting [34,35,36]. The experimental data collected for the HTM-LTM mix physical properties in terms of moisture content were used to develop regression models. 

## 3. Results and Discussion

**Figure 6:**
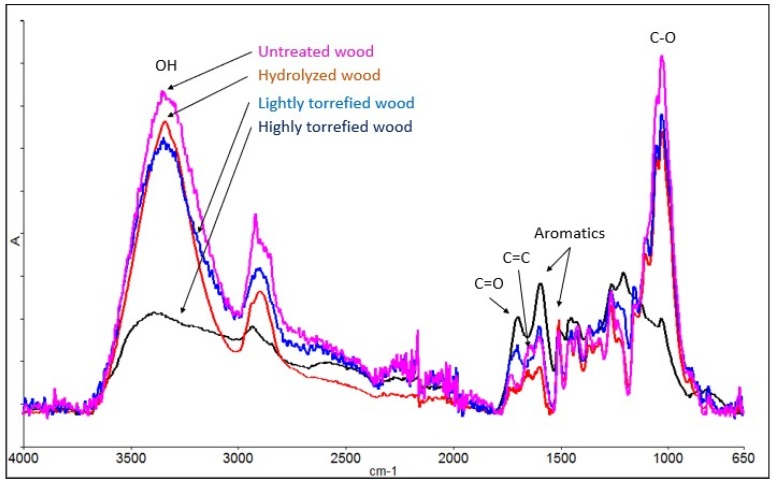
FTIR spectrums of the treated biomass used in this work.

Figure 6 shows the FTIR analysis performed for wood samples with mild torrefaction treatment to understand the in-situ lignin binding during briquetting. The results show a decreasing trend of OH (~3400 cm^−1^) groups for the treated biomass compared to the untreated biomass, which indicates the increasing hydrophobicity with the torrefaction treatments. C–O (~1050 cm^−1^) also showed a similar trend; however, for lightly torrefied treatments, the intensities were similar. Substituted aromatics (1600 cm^−1^) increased for highly torrefied biomass whereas for the lightly torrefied biomass the aromatics increase was marginal, which is due to the aromatic ring substitution that occurs in the process of torrefaction. The lowest substituted aromatics in lightly torrefied material indicate the highest reactivity in lignin and thus, the highest binding ability compared to other types of wood samples used in this study. It is also worthy to note that all types of treatments reduced C–H groups and reduced the aliphatic nature of the wood. 

Table 3 shows the characterization results for the produced briquettes. The data reported is an average of two measurements. As shown in Table 3, the briquette with 10.75% moisture performed better than the other samples. They showed a higher durability of 68% and the lowest water gain after 30 s as well as after 30 min, when compared to higher moisture-containing briquettes. 

### 3.1. Impact of LTM Material and Moisture Content on Briquette’s Shape and Binding

Our processing conditions of 225–250 °C temperature and 10,000-psi pressure and steam were the main activating parameters to release lignin for binding. With an increase in the moisture content of the mixture, the resulting briquettes became darker, developed a shiny cake layer on the briquette, and showed an increased number of cracks. The amount of steam released with the briquettes also increased. In most of the cases, the cake layer was fragile and peeled off easily. The die temperature was also reduced from 250–175 °C with the high moisture content in the mix and the steam it generated.

**Figure 7:**
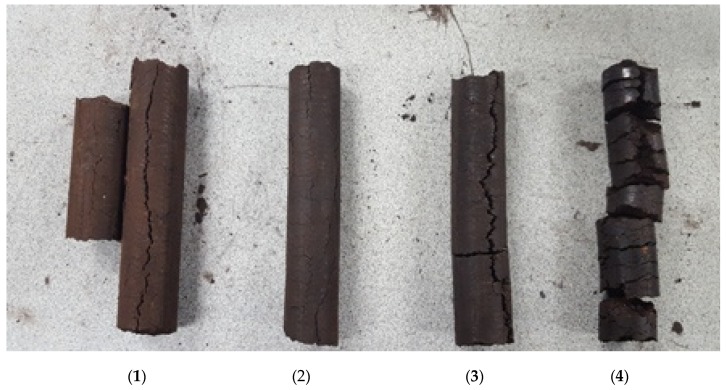
Produced briquettes (**1**) LTM, (**2**) mix-10.75% moisture, (**3**) mix-14% moisture, and (**4**) mix-16% moisture.

As shown in Table 3, for moisture content above 10.75%, a reduction in the durability of the briquette was observed. In addition, as shown in Figure 7, a high level of moisture content affected the appearance and shape of briquettes. It appeared that the combination of moisture, temperature, and pressure did mobilize the lignin—as evidenced by the darkness and hydrophobicity seen radially through the briquette. Perhaps, at higher moisture content, a hydrothermal treatment of wood was simulated. 

HTM, LTM, and the promoted steam (hydrothermal) treatment made the briquettes darker. The shiny appearance in the surface of the briquette may refer to the plastic deformation of particles and inherent binders that are squeezed out of the biomass cells due to high amounts of moisture, high temperature, and the application of pressure during densification. The moisture content in the HTM-LTM mixer resulted in steam and appeared to promote lubrication along the die walls. The pressure and frictional heat generated during extrusion appeared to soften and mobilize the lignin in the HTM-LTM mix. Adding LTM and increasing the moisture content to 10.75% can produce briquettes with a decent shape; however, after increasing the moisture content above 10.75%, the briquettes appeared darker and less dense. The high moisture and the resulting steam formation during densification led to a significant number of cracks, and this divided the briquettes into small round pucks with cracks. Tumuluru et al. [36], in their studies, indicated that densification of biomass using pelleting at higher moisture contents results in moisture flash-off and produces an expanded product. The expansion results in a lowering of the density of the densified product. In addition, the escape of moisture through the densified product results in crack formation. In the present study, the diameter measurement results in Table 3 show that the briquettes expanded compared to the inner diameter of the briquette press die. The LTM briquettes expanded by about 2 mm while the mixed briquettes with higher moisture contents expanded a little more than 2 mm due to cracks. 

### 3.2. Unit Density

**Figure 8:**
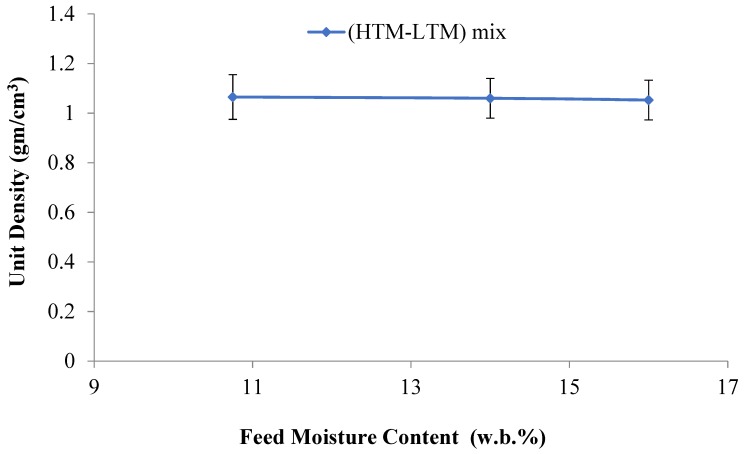
Unit density vs feed moisture content.

**Figure 9:**
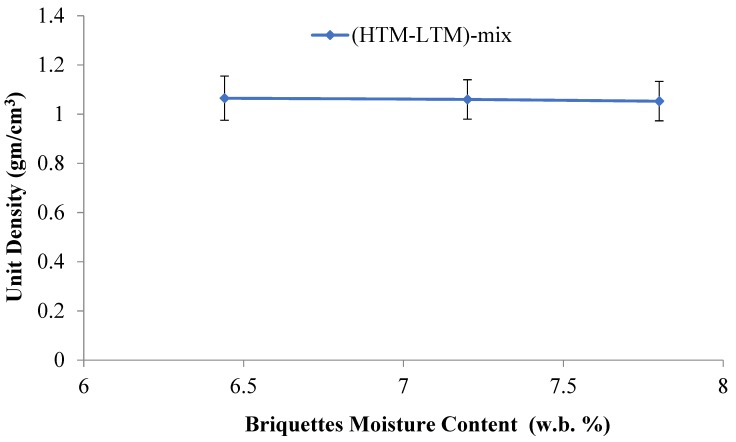
Impact of briquette moisture content on the bulk density.

Unit density is an important characteristic of biomass briquettes due to its direct influence on the handling and transportation of materials. According to the results in Table 3, unit density decreases when HTM materials are added and the moisture content is increased. This is due to the lower density of the HTM when compared to the LTM. Also, the decreased unit density values in the mixed HTM-LTM briquettes is strongly related to the resulting shape, cracks, and initial moisture content in the raw or mix materials. In their review on the biomass densification, Tumuluru et al. [37] suggested that the particle size, shape, and moisture content of biomass have a significant effect on the unit and bulk density of the densified products. A recent study by Tumuluru [38] on the pelleting characteristics of pine and switchgrass blends indicated that the bulk density of blended pellets is lower compared to pellets made using pure pine and higher compared to pure switchgrass pellets. The author reasoned that the change in particle size by blending resulted in changes in the bulk density of the blended pellets. The lower unit density values at higher moisture contents in the mixed briquettes agrees with the work of Tumuluru et al. on other materials [39]. This is shown in Figure 8, in which the unit density decreases with the increasing feed moisture content in the tested samples taken from the produced briquettes. Nevertheless, the differences in the values are small and are considered acceptable. The highest resulting unit density was found for the 10.75% moisture briquettes, which was evident by more compaction, higher binding, and lower cracks. Figure 9, which is drawn for briquette moisture content to unit density, indicates that an increase in briquette moisture causes the unit density of the briquette to decrease. 

### 3.3. Hydrophobicity

**Figure 10:**
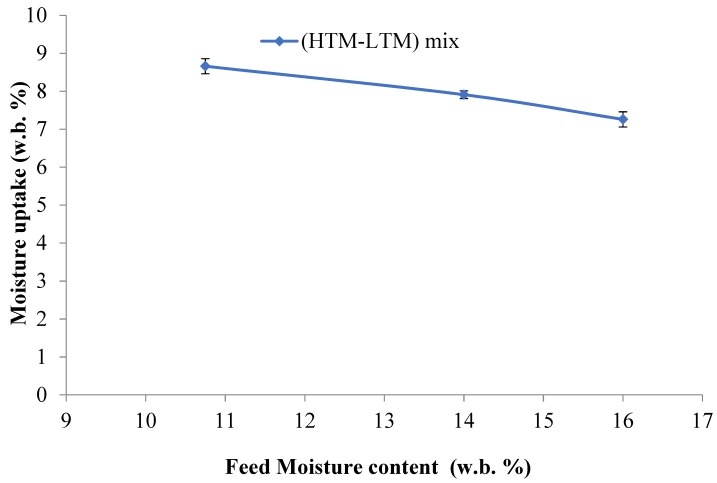
Moisture uptake in the briquettes at 90% relative humidity, 30 °C storage for a 24-h storage period.

**Figure 11:**
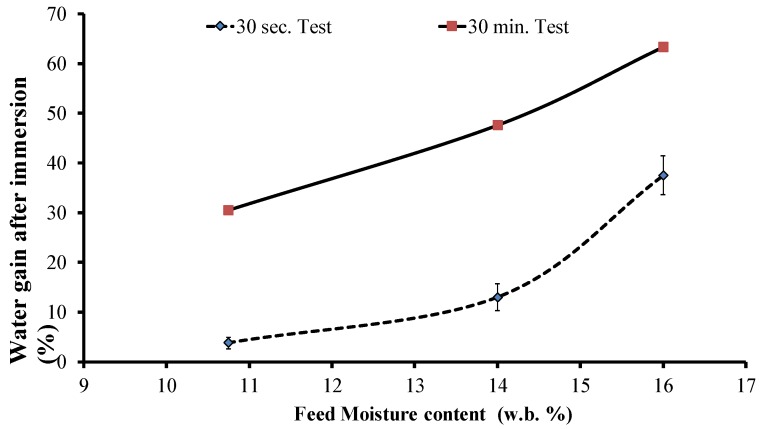
Water gain after immersion vs. feed moisture content for two test periods.

**Figure 12:**
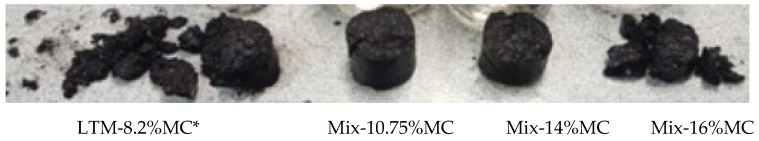
Water immersion results. *MC: Moisture content.

**Figure 13:**
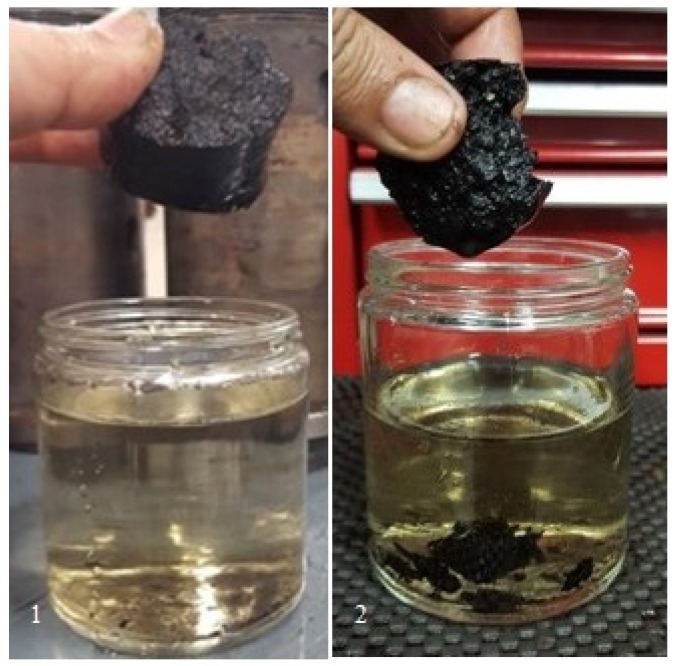
After 24 h of water immersion: (**1**) 10.75% moisture, (**2**) 16% moisture.

Briquette exposure to humid or rainy environments during transportation and storage could adversely affect its quality [30]; hence, evaluating the hydrophobicity of the briquettes is important. As can be seen in Table 3 and Figure 10, the moisture uptake numbers reveal that it decreased with increased feed moisture content, which indicates that increasing the moisture content in feed does improve hydrophobicity. However, for the water immersion results, the situation is the opposite, in which higher feed moisture led to increased water uptake (Figure 11). Since this test uses the direct contact of water, it depends more on the briquette’s quality and the number of cracks in it. At 30 s, the mixed briquette with 10.75% moisture gave the lowest water uptake followed by the LTM briquette. Increasing the immersion time to 30 min still showed that the 10.75% moisture content sample had the lowest uptake, while the LTM sample absorbed more water than all the mixed briquettes and eventually disintegrated, as shown in Figure 12. The cracks that are formed during the briquetting process might result in more moisture uptake, as shown by the water gain after 30 s and 30 min during the water immersion tests (Table 3). Further, the 10.75% and 14% moisture content samples stayed intact even at the end of this test. Figure 13 shows the 24-hour water immersion test results, which revealed that the moisture content of the 10.75% sample had increased water resistance and the sample stayed solid and intact after taking it out from water. In fact, the 10.75% sample stayed intact even after 10 days of soaking. The briquette sample with 16% moisture broke easily when pressed slightly by hand. Although a higher moisture content can provide these briquettes with enhanced binding, as discussed above, moisture content >11% in the HTM+LTM mix did not help in binding the whole briquettes together along the die. This is evidenced by the increased number of cracks and breaks in the briquettes during the release of excessive moisture as steam. 

### 3.4. Durability 

**Figure 14:**
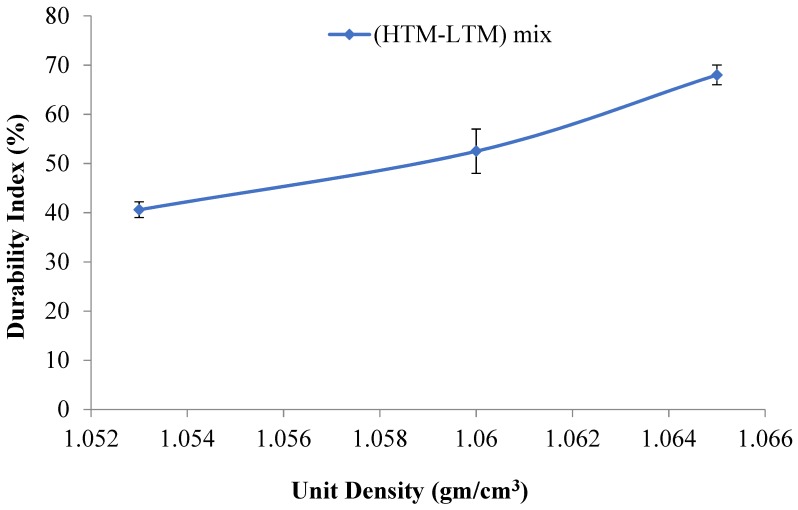
Linear correlation between mechanical durability and unit density.

**Figure 15:**
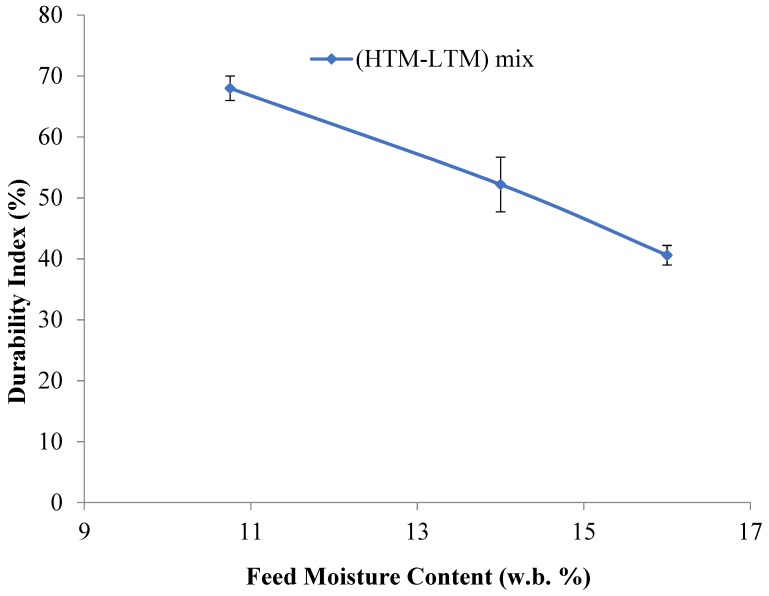
Durability versus feed moisture content for the HTM-LTM mixtures.

Mechanical durability is one of the significant parameters from the viewpoint of handling and transportation when utilizing existing coal infrastructure. The durability index results of each sample are shown in Table 3. The highest durability index was found for the HTM-LTM mix sample with a 10% to 11% moisture content. At this moisture content, the briquettes showed fewer cracks and enhanced overall binding (i.e., mechanical binding) along with binding promoted by the inherent lignin. The increase in durability for this HTM-LTM mix compared to the LTM materials might be due to in-situ lignin mobility created by the combined effect of feed moisture content, temperature, pressure, and steam generation that occurred inside the die. Figure 14 shows a plot of the measured mechanical durability values versus the calculated unit density values and this illustrates a good linear correlation between the durability index and unit density of the briquettes produced using the HTM + LTM mix. These trends indicate that percent durability increased with the increase in the unit density of the briquettes produced. This might be due to better mechanical particle interlocking, as well as binding by the mobilized in-situ lignin in the biomass. While a higher moisture content plays a significant role in the activation and mobility of in-situ lignin with the help of the generated steam, excess steam generation can produce the opposite effect. Figure 15 shows that for high-moisture cases, binding activated by in-situ lignin was not enough to overcome the cracks initiated by steam (generated inside the die by the excessive moisture content) upon the release of briquettes from the die. Based on these results, we expect that torrefaction of the LTM material at lower temperatures of 200 °C or less, instead of 240 °C as the tests in this research, can help to improve the durability of the briquettes, as the intrinsic binding ability of the lignin might be preserved. 

### 3.5. Energy Density

As shown in Table 3, the briquette made with 100% LTM had a lower energy content compared to HTM-LTM mix briquettes. The addition of HTM not only improved the energy content of the briquette but also helped improve the hydrophobicity. According to Table 3, a higher calorific value directly relates to the use of highly torrefied materials (HTMs). On the other hand, the higher moisture content helped in raising the heating value by promoting simulated hydrothermal cooking (i.e., by steam and pressure) inside the briquette press die during densification even though these conditions resulted in briquettes with less durability due to cracks that formed because of flash-off of moisture once the briquettes exited the die. 

### 3.6. Data Analysis

Regression equations of the experimental data developed for the moisture content and the product properties for HTM-LTM mix are illustrated in Table 4. Coefficient of determination values (R^2^) higher than 0.95 indicate the models adequately described the product properties with respect to the moisture content. The unit density of the briquette is negatively related to the feed and briquette moisture content. In the case of moisture uptake, it is also negatively correlated to the moisture content of feed materials. The durability of the briquettes produced is positively correlated to the unit density of the briquettes while the feed moisture content had a positive impact up to a certain level (12%, w.b.), whereas further increasing the feed moisture content above 12% (w.b.) had a negative impact on the durability of the briquettes produced. 

### 3.7. Fourier Transform Infrared Spectroscopy (FTIR)

**Figure 16:**
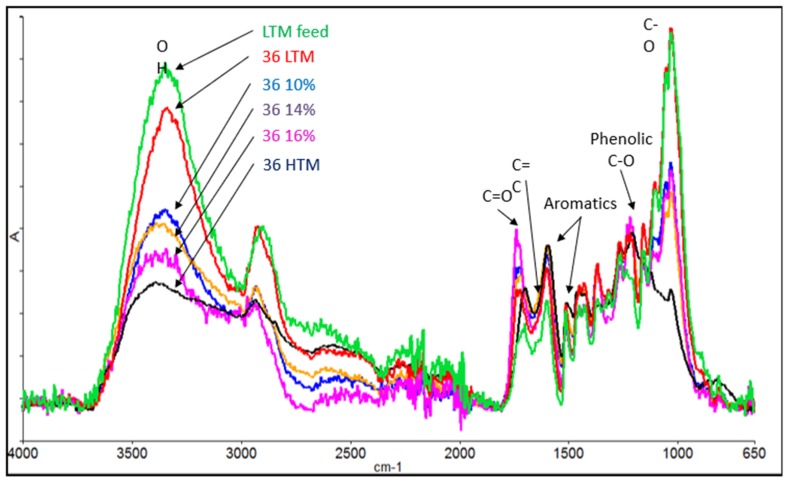
FTIR spectrums of the densified biomass samples.

According to Figure 16, increasing the moisture content in the feed reduced the oxygen bearing functional groups, such as O–H (3350 cm^−1^) and C–O (1050 cm^−1^). This observation is consistent with the higher hydrophobicity observed for high moisture runs in the hydrophobicity chamber test. This indicates that steam generated during densification (due to the available moisture) can possibly act as a dehydrating/deoxygenating agent, reducing these O–H and C–O bonds. The LTM feed had the lowest number of substituted aromatics (1600 cm^−1^) and phenolic C–O bonds (1250 cm^−1^), indicating a high binding ability, and these peaks was significantly increased in the densified briquettes with cross-linking reactions. Aliphatic CH (2950 cm^−1^) bonds were also reduced in the higher moisture runs due to the reduction of weaker aliphatic carbon, such as hemicellulose, in the biomass. C=O is the highest and lowest in 36% to 16% and LTM feed, respectively, and this indicates that these groups increase with increasing moisture in briquetting, probably due to cleavage of the C–O–C bonds in lignin and carbohydrate polymers. This indicates that a certain amount of depolymerization also occurs during briquetting. However, C=O is lower in 36-HTM compared to —16−36% due to decarbonylating occurring in severe torrefaction conditions in HTM.

## 4. Conclusions

The results showed that a lightly torrefied material (LTM) can act as a binder during briquetting of highly torrefied material (HTM). Mixing LTM with HTM material in combination with moisture, temperature, and pressure resulted in improving the durability and hydrophobicity of the briquettes. During briquetting and due to the applied pressure, the migration of lignin and extractives to the surface of briquettes was promoted with the help of steam generated due to the added moisture. The migration also promoted the formation of a wax-like coating and a shiny appearance to the briquettes produced. The lignin migration during briquetting process acted as in-situ binding of the biomass particles and in turn, improved the briquette properties, such as unit density, durability, and hydrophobicity. As an example, for briquettes with 10% to 11% moisture and a 20:80 LTM to HTM ratio, the calorific value and durability increased by 266 BTU/lb and 42%, respectively, compared to LTM-only briquettes. For the same briquettes, water absorption was reduced by 57% compared to LTM briquettes. This study indicates that there is a strong correlation between feed moisture content and briquette durability. From this study, we can conclude that lightly torrefied material can be used as a source of natural binders (lignin) as it provides the relatively less-modified lignin that is necessary for binding, while highly torrefied biomass provides a higher hydrophobicity and calorific value. 

## Figures and Tables

**Table 1:** Characterization of feed materials.

Material	LTM	HTM
Moisture Content (%, w.b.)	6.8–7.2	5.4–6.7
Chip particle size (length in mm)	1–6	1–20
Heating Value (BTU/LB)	9050–9064	9490–9560

**Table 2:** Moisture content of the torrefied material used for briquetting tests.

Expt. No	Material	Feed Moisture Content (%, w.b.)
1	LTM	8.2
2	HTM-LTM	10.75
3	HTM-LTM	14
4	HTM-LTM	16

Note: LTM: Lightly torrefied material, HTM: Highly torrefied material.

**Table 3:** Characterization results (average) for briquettes produced using 36-mm-diameter die.

Material		LTM	20% HTM-LTM 10%-MC	20% HTM-LTM 14%-MC	20% HTM-LTM 16%-MC
Briquetting process conditions					
Moisture content (%)		8 ± 0.2	10 ± 0.75	14 ± 0.3	16 ± 0.2
Briquette press die diameter (mm)		36	36	36	36
Briquette properties					
Briquette Diameter (mm)		38	38	38.5	38.5
Briquette Unit Density (gm/cm^3^)		1.178 ± 0.09	1.065 ± 0.09	1.060 ± 0.08	1.053 ± 0.08
Moisture content of produced briquettes (%)		6.31 ± 0.25	6.44 ± 0.93	7.2 ± 0.08	7.8 ± 0.43
Moisture uptake (%)	RH—90%, Temperature—30 °C, Time—24 h	8.23 ± 0.25	8.66 ± 0.2	7.91 ± 0.1	7.26 ± 0.2
Water gain after immersion (%)	30 s test	7.2 ± 1.9	3.8 ± 1.19	13 ± 2.7	37.5 ± 3.9
	30 min test	69.8 ± 0.7	30.5 ± 0.2	47.6 ± 0.6	63.3 ± 0.6
Durability Index (%)		44.6 ± 1.1	68 ± 2	52.5 ± 4.5	40.6 ± 1.6
Energy Density BTU/lb KJ/Kg		9103 ± 53 21,173.6	9369 ± 3 21,792.3	9385 ± 18 21829.5	9459 ± 44 22,001.6

**Table 4:** Regression equations of the experimental data.

Product Property		Regression Model	Coefficient of Determination Value (R^2^)
Unit Density (y) (gm/cm^3^) Versus Feed Moisture Content (*x*) (%)		y = −0.002*x* + 1.089	0.946
Unit Density (y) (gm/cm^3^) Versus Briquette Moisture Content (*x*) (%)		y = −0.0087*x* + 1.1217	0.97
Moisture Uptake (y) (%) Versus Feed Moisture Content (%) (*x*)		y = −0.263*x* + 11.51	0.990
Water Gain (y) (%) Versus Feed Moisture Content (%) (*x*)	30 s. Test	y = 6.073*x* − 64.39	0.853
	30 Min. Test	y = 6.152*x* − 36.43	0.987
Durability Index (%) (y) Versus Unit Density (gm/cm^3^) (*x*)		y = 2245x − 2325	0.970
Durability Index (%) (y) Versus Feed Moisture Content (%) (*x*)		y = −5.184*x* + 124.0	0.997

## References

[1] Tumuluru J.S., Hess J.R., Boardman R.D., Wright C.T., Westover T.L. (2012). Formulation, pretreatment, and densification options to improve biomass specifications for co-firing high percentages with coal. Ind. Biotechnol..

[2] Tag A.T., Dumana G., Ucarb S., Yanika J. (2016). Effects of feedstock type and pyrolysis temperature on potential applications of biochar. J. Anal. Appl. Pyrolysis.

[3] Wang C., Peng J., Li H., Bi X.T., Legros R., Lim C.J., Sokhansanj S. (2013). Oxidative torrefaction of biomass residues and densification of torrefied sawdust to pellets. Bioresour. Technol..

[4] Bridgeman T.G., Jones J.M., Shield I., Williams P.T. (2008). Torrefaction of reed canary grass, wheat straw and willow to enhance solid fuel qualities and combustion properties. Fuel.

[5] Yang Z., Sarkar M., Kumar A., Tumuluru J.S., Huhnke R.L. (2014). Effects of torrefaction and densification on switchgrass pyrolysis Products. Bioresour. Technol..

[6] Anukam A., Mamphweli S., Reddy P., Okoh O., Meyer E. (2015). An Investigation into the Impact of Reaction Temperature on Various Parameters during Torrefaction of Sugarcane Bagasse Relevant to Gasification, Hindawi Publishing Corporation. J. Chem..

[7] Asadullah M., Adi A.M., Suhada N., Malek N., Saringat M.I., Azdarpour A. (2014). Optimization of palm kernel shell torrefaction to produce energy densified bio-coal. Energy Convers. Manag..

[8] Chen W.H., Peng J., Bi X.T. (2015). A state-of-the-art review of biomass torrefaction, densification and applications. Renew. Sustain. Energy Rev..

[9] Tumuluru J.S., Sokhansanj S., Hess J.R., Wright C.T., Boardman R.D. (2011). A review on biomass torrefaction process and product properties for energy applications. Ind. Biotechnol..

[10] Rodrigues T.O., Rousset P.L.A. (2009). Effects of torrefaction on energy properties of eucalyptus grandis wood. CERNE.

[11] Ghiasi B., Kumar L., Furubayashi T., Lim C.J., Bi X., Kim C.S., Sokhansanj S. (2014). Densified biocoal from woodchips: Is it better to do torrefaction before or after densification?. Appl. Energy.

[12] Guo L., Wang D., Tabil L.G., Wang G. (2016). Compression and relaxation properties of selected biomass for briquetting. Biosyst. Eng..

[13] Nanou P., Huijgen W.J.J., Carbo M.C., Kiel J.H.A. (2018). The role of lignin in the densification of torrefied wood in relation to the final product properties. Biomass Bioenergy.

[14] Brodin I. (2009). Chemical Properties and Thermal Behavior of Kraft Lignins. Ph.D. Thesis.

[15] Peng J., Bi X.T., Lim C.J., Peng H., Kim C.S., Jia D., Zuo H. (2015). Sawdust as an effective binder for making torrefied pellets. Appl. Energy.

[16] Peng J., Wang J., Bi X.T., Lim C.J., Sokhansanj S., Peng H., Jia D. (2015). Effects of thermal treatment on energy density and hardness of torrefied wood pellets. Fuel Process. Technol..

[17] Muazu R.I., Stegemann J.A. (2017). Biosolids and microalgae as alternative binders for biomass fuel briquetting. Fuel.

[18] Peng J.H., Bi X.T., Sokhansanj S., Lim C.J. (2013). Torrefaction and densification of different species of softwood residues. Fuel.

[19] Li H., Liu X., Legros R., Bi X.T., Lim C.J., Sokhansanj S. (2012). Pelletization of torrefied sawdust and properties of torrefied pellets. Appl. Energy.

[20] Larsson S.H., Rudolfsson M., Nordwaeger M., Olofsson I., Samuelsson R. (2013). Effects of moisture content, torrefaction temperature, and die temperature in pilot scale pelletizing of torrefied Norway spruce. Appl. Energy.

[21] Araújo S., Boas M.A.V., Neiva D.M., de Cassia Carneiro A., Vital B., Breguez M., Pereira H. (2016). Effect of a mild torrefaction for production of eucalypt wood briquettes under different compression pressures. Biomass Bioenergy.

[22] Järvinen T., Agar D. (2014). Experimentally determined storage and handling properties of fuel pellets made from torrefied whole-tree pine chips, logging residues and beech stem wood. Fuel.

[23] Hu Q., Shao J., Yang H., Yao D., Wang X., Chen H. (2015). Effects of binders on the properties of bio-char pellets. Appl. Energy.

[24] Kong L., Tian S., Li Z., Luo R., Chen D., Tu Y., Xiong Y. (2013). Conversion of recycled sawdust into high HHV and low NOx emission bio-char pellets using lignin and calcium hydroxide blended binders. Renew. Energy.

[25] Zhong Q., Yang Y., Li Q., Xu B., Jiang T. (2017). Coal tar pitch and molasses blended binder for production of formed coal briquettes from high volatile coal. Fuel Process. Technol..

[26] Teixeira S.R., Pena A.F.V., Miguel A.G. (2010). Briquetting of charcoal from sugar-cane bagasse fly ash (scbfa) as an alternative fuel. Waste Manag..

[27] Kaliyan N., Vance Morey R. (2010). Natural binders and solid bridge type binding mechanisms in briquettes and pellets made from corn stover and switchgrass. Bioresour. Technol..

[28] Anna C.S., Souza W.D., Méndez-Vilas A. (2012). Microscopy as a tool to follow deconstruction of lignocellulosic biomass. Current Microscopy Contributions to Advances in Science and Technology.

[29] Bhagwanrao S.V., Singaravelu M. (2014). Bulk density of biomass and particle density of their briquettes. Int. J. Agricultural Eng..

[30] Kaliyan N., Vance Morey R. (2009). Factors affecting strength and durability of densified biomass products. Biomass Bioenergy.

[31] Bazargan A., Rough S.L., McKay G. (2014). Compaction of palm kernel shell biochars for application as solid fuel. Biomass Bioenergy.

[32] Temmerman M., Rabier F., Jensen P.D., Hartmann H., Böhm T. (2006). Comparative study of durability test methods for pellets and briquettes. Biomass Bioenergy.

[33] ISO 15210 (2011). Solid Biofuels—Determination of Mechanical Durability of Pellets and Briquettes.

[34] Miao Z., Grift T.T., Hansen A.C. (2011). Energy requirement for comminution of biomass in relation to particle physical properties. Ind. Crop. Prod..

[35] Tumuluru J.S. (2014). Effect of the process variables on the density and durability of the pellets made from high moisture corn stover. Biosyst. Eng..

[36] Tumuluru J.S. (2016). Specific energy consumption and quality of the wood pellets produced using high moisture lodgepole pine grind in a flat die pellet mill. Chem. Eng. Res. Des..

[37] Tumuluru J.S., Wright C.T., Hess J.R., Kenney K.L. (2011). A Review on Biomass Densification Systems to Develop Uniform Feedstock Commodities for Bioenergy Application. Biofuels. Bioprod. Biorefining.

[38] Tumuluru J.S. (2019). Pelleting of Pine and Switchgrass Blends: Effect of Process Variables and Blend Ratio on the Pellet Quality and Energy Consumption. Energies.

[39] Tumuluru J.S., Tabil L.G., Song Y., Iroba K.L. (2015). Impact of process conditions on the density and durability of wheat, oat, canola, and barley straw briquettes. Bioenergy Res..

